# Archaeal replicative primase mediates DNA double-strand break repair

**DOI:** 10.1093/nar/gkaf322

**Published:** 2025-04-24

**Authors:** Daijiang Xiong, Zhimeng Li, Wen Qi, Shaoying Wang, Junkai Huang, Ningning Zhang, Zhenfeng Zhang, Li Huang

**Affiliations:** State Key Laboratory of Microbial Resources, Institute of Microbiology, Chinese Academy of Sciences, No. 1 West Beichen Road, Chaoyang District, Beijing 100101, China; College of Life Science, University of Chinese Academy of Sciences, Beijing100049, China; Southern Marine Science and Engineering Guangdong Laboratory (Guangzhou), Nansha, Guangzhou 511458, China; State Key Laboratory of Microbial Resources, Institute of Microbiology, Chinese Academy of Sciences, No. 1 West Beichen Road, Chaoyang District, Beijing 100101, China; College of Life Science, University of Chinese Academy of Sciences, Beijing100049, China; State Key Laboratory of Microbial Resources, Institute of Microbiology, Chinese Academy of Sciences, No. 1 West Beichen Road, Chaoyang District, Beijing 100101, China; College of Life Science, University of Chinese Academy of Sciences, Beijing100049, China; Southern Marine Science and Engineering Guangdong Laboratory (Guangzhou), Nansha, Guangzhou 511458, China; State Key Laboratory of Microbial Resources, Institute of Microbiology, Chinese Academy of Sciences, No. 1 West Beichen Road, Chaoyang District, Beijing 100101, China; College of Life Science, University of Chinese Academy of Sciences, Beijing100049, China; State Key Laboratory of Microbial Resources, Institute of Microbiology, Chinese Academy of Sciences, No. 1 West Beichen Road, Chaoyang District, Beijing 100101, China; College of Life Science, University of Chinese Academy of Sciences, Beijing100049, China; State Key Laboratory of Microbial Resources, Institute of Microbiology, Chinese Academy of Sciences, No. 1 West Beichen Road, Chaoyang District, Beijing 100101, China; College of Life Science, University of Chinese Academy of Sciences, Beijing100049, China; Southern Marine Science and Engineering Guangdong Laboratory (Guangzhou), Nansha, Guangzhou 511458, China

## Abstract

Archaea, often thriving in extreme habitats, are believed to have evolved efficient DNA repair pathways to cope with constant insults to their genomes. However, how these organisms repair DNA double-strand breaks (DSBs), the most lethal DNA lesions, remains unclear. Here, we show that replicative primase consisting of the catalytic subunit PriS and the noncatalytic subunits PriL and PriX from the hyperthermophilic archaeon *Saccharolobus islandicus* is involved in DSB repair. We show that the overproduction or knockdown of PriL increases or decreases, respectively, the rate of survival and mutation frequency of *S. islandicus cells* following treatment with a DNA damaging agent. The increase in mutation is attributed primarily to an increase in small insertions or deletions. Further, overproduction of PriL enhances the repair of CRISPR-generated DSBs *in vivo*. These results are consistent with the extraordinary ability of PriSL to promote annealing between DNA strands sharing microhomology in addition to the activity of the heterodimer in terminal transfer and primer extension. The primase-mediated DSB repair is cell-cycle dependent since PriL is barely detectable during the S/G2 transition. Our data demonstrate that replicative primase is involved in DSB repair through microhomology-mediated end joining in Archaea.

## Graphical Abstract

**Graphical Abstract:**
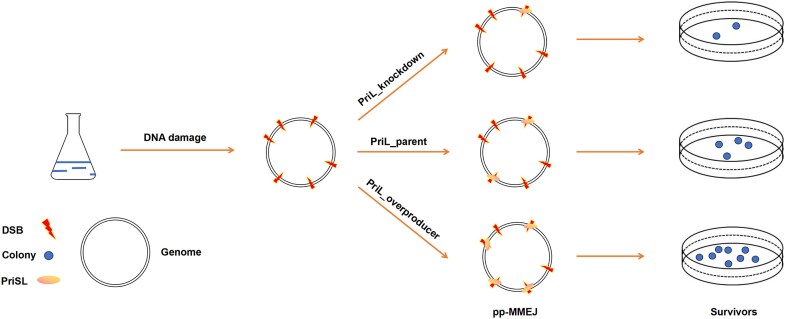

## Introduction

Initiation of DNA replication depends on primase for the *de novo* synthesis of short RNA primers, which are then extended by replicative DNA polymerases (Pols). Bacterial primase is a single multidomain protein, known as DnaG, which contains a TOPRIM fold within the catalytic domain [1–3]. Archaeal and eukaryotic primases consist of the catalytic subunit PriS or Pri1 and the regulatory subunit PriL or Pri2. The heterodimeric primase in eukaryotes forms a tight complex with Pol α (A/B) in a heterotetrameric assembly [4]. Both archaeal and eukaryotic primases are members of primase-polymerase (Prim-Pol) superfamilies, with homologues widely distributed across the three domains of life [5, 6]. Prim-Pols are structurally characterized by an N-terminal α/β fold and a C-terminal RNA recognition motif-like fold in the catalytic domain and biochemically characterized by the ability to catalyze both primer synthesis and polymerization [2, 5, 7, 8]. Despite sharing conserved structural features, Prim-Pols have evolved in different domains of life to serve various roles in DNA metabolism [5, 9].

The most extensively investigated role of Prim-Pols is the synthesis of primers during genome replication. PriS in complex with PriL in archaea or as part of the Pol α–primase complex in eukaryotes prime the synthesis of DNA, generating Okazaki fragments [10, 11]. However, there are distinct differences in biochemical characteristics between the archaeal and eukaryotic replicative primases. PriS from *Pyrococcus furiosus* and PriSL from several other archaea synthesize primers using both dNTPs and rNTPs [12–14], unlike eukaryotic Pri1, which synthesizes only RNA primers [15], but resemble eukaryotic Prim-Pol, which utilizes both rNTPs and dNTPs for primer synthesis [16]. In addition, archaeal primases are capable of generating long extension products (up to thousands of bases in length) as well as catalyzing template-independent terminal transfer [13, 17, 18]. The ability to perform both primer synthesis and strand extension allows Prim-Pols to function in more processes than primer synthesis. Notably, Prim-Pols play a role in DNA damage tolerance and DNA repair. Both archaeal and eukaryotic Prim-Pols are capable of translesion synthesis (TLS) as they were shown to bypass DNA lesions, such as 8-oxo-guanine and cyclobutane pyrimidine dimers [19–21]. Archaeal PriS has also been shown to replicate past deoxyuracils (dUs), even in the presence of stalled replicase complexes [21]. While all eukaryotes have Pri1, a Prim-Pol replicative primase, some also encode a second Prim-Pol, denoted as PrimPol. PrimPol can effectively reprime DNA synthesis downstream of ultraviolet (UV)-induced DNA damages, abasic sites, and cisplatin lesions [22–25]. Animal cells lacking PrimPol show increased sensitivity to UV radiation [23, 26]. Therefore, Prim-Pols provide a DNA damage tolerance mechanism to promote genome stability [27].

In bacteria, Prim-Pol orthologues are found co-operonically with Ku [28, 29], whose eukaryotic counterpart is responsible for binding to the ends of DNA double-strand breaks (DSBs) during nonhomologous end joining (NHEJ) repair. The Prim-Pol domain (PolDom/Prim-PolD) is part of a larger DSB repair protein known as ligase D (LigD), which also has DNA ligase (Lig), Pol, and phosphoesterase (PE) domains [30, 31]. LigD, along with Ku, is capable of NHEJ repair. This process of bacterial NHEJ repair is facilitated by the unusual ability of Prim-Pol to promote microhomology-mediated end joining (MMEJ) and subsequent gap filling [32, 33]. In addition, a Prim-PolD homologue called Prim-PolC is associated operonically with ligase C (LigC) in *mycobacteria*, which also encodes Prim-PolD [34]. Prim-PolC is involved in LigC-dependent repair of short DNA gaps produced during excision repair [35]. A DNA break repair complex highly homologous to the bacterial NHEJ apparatus is also found in the methogenic archaeon *Methanocella paludicola* [31]. The archaeal complex, which consists of DNA Lig, Pol, PE, and Ku, repairs DNA breaks using RNA intermediates. However, very few archaeal species possess a Ku protein, and no Ku-independent NHEJ pathways have been reported in Archaea, which often thrive in extraordinarily stressful environments, and yet do not appear to display increased rates of spontaneous mutation [36]. It is therefore of considerable interest to explore DNA repair systems in these organisms.

The replicative primase from hyperthermoacidophilic archaea of the order Sulfolobales has been extensively studied. We showed previously that the Prim-Pol primase from *Saccharolobus solfataricus* (formerly known as *Sulfolobus solfataricus*) consists of three subunits, i.e. PriS, PriL, and the second regulatory subunit PriX, which form a heterotrimer of the 1:1:1 ratio *in vitro* [37]. All three subunits are essential for the viability of the organism. PriL contains a highly conserved Fe–S cluster in its C-terminal domain (PriL-CTD). PriX is a diverged homolog of the PriL-CTD but lacks the Fe–S cluster. Intriguingly, the molar ratio of PriX, PriL, and PriS is ∼1:2:13 *in vivo* [37], suggesting the presence of various forms of complexes including PriSLX and PriSL. While both PriSLX and PriSL are active in primer synthesis, the former is over an order of magnitude more efficient than the latter in the reaction. Further, PriL, but not PriX, enhances primer extension by PriS, whereas PriX is specifically involved in the initiation step of primer synthesis [37]. Intriguingly, PriS, alongwith a chimeric protein constructed by fusing PriL’s N-terminal domain (PriL-NTD) with N-terminally truncated PriX, is nearly as active as PriSLX, indicating that the Fe–S cluster is not essential for primer synthesis *in vitro* [38]. Notably, PriSL promotes efficient annealing between DNA strands sharing microhomology with as short as only two GC pairs, and is capable of template-dependent polymerization across discontinuous templates (PADT) [17]. This observation and the structural resemblance of PriS to the Pol domain of bacterial LigD [39] prompted the speculation that the protein complex may play a role in DSB repair.

In this study, we show that overproduction of PriL increases the rate of survival and mutation frequency (MF) of *Saccharolobus islandicus* following DNA damage treatment which generates DSBs, and further demonstrate that PriSL mediates DSB repair through MMEJ.

## Materials and methods

### Growth of *Saccharolobus* strains

*Saccharolobus islandicus* E233S (Δ*pyrEF*Δ*lacS*) and its derivative strains were grown with shaking at 75°C in basic salts supplemented with 0.2% sucrose, 0.2% casamino acids, and vitamins (SCV), SCV supplemented with 0.005% yeast extract and 20 μg/ml uracil (SCVyU), or SCV with D-arabinose replacing sucrose (ACV), or on 0.8% gelrite plates containing the above medium [40, 41].

### Strain construction

An *S. islandicus* strain encoding a chimeric protein consisting of the PriL-NTD and the N-terminally truncated PriX, whose synthesis was under the control of the native promoter of *priL*, was constructed by following a gene editing protocol described previously [42] with modifications. A 40-bp target spacer sequence from the *priL* gene was prepared by annealing oligonucleotides Lspa-F and Lspa-R (Supplementary Table S1) and inserted into the BspMI site of the IA-type CRISPR gene editing plasmid pSe-Rp (protospacer adjacent motif (PAM): 5′-CCN-3′). Two sequences flanking the portion of the gene encoding the PriL-CTD (residues 212–307), i.e. the left and right homologous arms (∼600 bp each), were prepared by polymerase chain reaction (PCR) using primer pairs L_L_-SalI-F/L_SOE_-R and L_SOE_-F/L_R_-NotI-R, respectively (Supplementary Table S1). The sequence encoding the N-terminally truncated PriX (residues 42–154) was amplified by PCR with primers Xc-F and Xc-R. The two flanking sequences were then joined with the partial *priX* sequence through overlap extension PCR to give rise to a fragment coding for the chimeric protein PriLn-Xc. This fragment was inserted between the SalI and NotI sites of pSe-Rp containing the target spacer. The resulting plasmid was electroporated into strain E233S. After growth on plates, colonies were picked, and those containing the gene encoding the chimeric protein were identified and purified. The strain is denoted CHI. To delete the PriX gene from strain CHI, a 40-bp target spacer sequence from *priX* was prepared by annealing oligonucleotides Xspa-F and Xspa-R (Supplementary Table S1), and inserted into the BspMI site of pSe-Rp. Two fragments containing sequences flanking *priX* (∼600 bp each) were prepared by PCR using primers XL-SalI-F/XSOE-R and XSOE-F/XR-SalI-R, respectively (Supplementary Table S1), and inserted between the SalI and NotI sites of pSe-Rp containing the target spacer. The plasmid was electroporated into CHI. After growth on plates, colonies with the *priX* deletion were identified and purified. The resulting strain was termed CHI-ΔX.

A PriL knockdown strain (PriL-kd) was constructed by inserting a 40-bp *priL*-targeting spacer sequence, prepared by annealing oligonucleotides Lkd-spa-F and Lkd-spa-R (Supplementary Table S1), into the BspMI site of the IIIB-type CRISPR gene editing plasmid pAC (PAM: 5′-GAAAG-3′) [43]. The plasmid was subsequently introduced into strain E233S by electroporation.

A PriL overproducing strain (PriL-op) was constructed by inserting a *priL* sequence, prepared by PCR using primer pairs Lop-F/Lop-R (Supplementary Table S1), between the SalI and NotI restriction sites of expression plasmid pSeSD [40] and transforming strain E233S with the plasmid.

### Protein preparation

To construct a PriSLX co-expression plasmid, *priS*, *priL*, and *priX* were amplified by PCR from the genomic DNA of *S. islandicus* E233S using primer pairs S-BamHI-F/S-NotI-R, L-NdeI-F/L-XhoI-R, and X-NdeI-F/X-XhoI-R, respectively (Supplementary Table S1). The *priS* and *priL* fragments were inserted into the BamHI/NotI and NdeI/XhoI sites, respectively, of plasmid pETDuet (EMD Biosciences), and the *priX* fragment was inserted into the NdeI/XhoI sites of plasmid pRSFDuet (EMD Biosciences). The two plasmids were co-transformed into Rosetta2 (DE3) cells (TransGene), yielding a PriSLX overproducer. To overproduce a PriSLn-Xc co-expression plasmid, the *priLn-Xc* fragment was obtained from the genomic DNA of CHI-ΔX by PCR using primers Ln-Xc-BamHI-F and Ln-Xc-NotI-R (Supplementary Table S1). *priS* was amplified by PCR using the primers S-NdeI-F/S-XhoI-R. The *priLn-Xc* and *priS* fragments were inserted into the BamHI/NotI and NdeI/XhoI sites, respectively, of plasmid pETDuet. The expression vectors were introduced into Rosetta2 (DE3) cells by chemical transformation (TransGene).

To prepare recombinant PriSLX and PriSLn-Xc, the above overproducer strains were grown to the exponential phase, and the synthesis of the proteins was induced by the addition of 0.8 mM isopropyl β-D-thiogalactoside (IPTG). Cells were harvested and sonicated in buffer A [30 mM Tris–HCl (pH 8.0), 500 mM NaCl, 20 mM imidazole, and 10% (w/v) glycerol]. The lysate was centrifuged at 17 000 × *g* for 30 min at 4°C. The supernatant was heat-treated at 70°C for 20 min, and centrifuged again at 17 000 × *g* for 30 min at 4°C. Polyethylenimine was added to the supernatant to a final concentration of 0.5% to remove DNA. After centrifugation at 17 000 × *g* for 30 min at 4°C, the supernatant was dialyzed against buffer A. The dialysate was filtered through a 0.22-μm filter membrane (1 ml, GE Healthcare) and loaded onto a Histrap column (1 ml, GE Healthcare) pre-equilibrated with buffer A. The target protein was eluted with an imidazole gradient (0–500 mM) in buffer A. Fractions containing the target protein were pooled and dialyzed against buffer B [30 mM Tris–HCl (pH 8.0), 100 mM NaCl, and 10% (w/v) glycerol]. The sample was loaded onto a Heparin SP column (1 ml, GE Healthcare) pre-equilibrated with buffer B. The bound proteins were eluted with a NaCl gradient (0.1–1 M) in buffer B. Peak fractions containing the pure target protein were pooled and concentrated by ultrafiltration. The purified protein was stored in buffer C [30 mM Tris–HCl (pH 8.0), 50 mM NaCl, and 10% (w/v) glycerol]. Protein concentration was determined by the Lowry method.

### Cell-cycle analysis

*Saccharolobus islandicus* strains were grown to an OD_600_ of ∼0.2, and treated for 6 h with 6 mM acetic acid. Cells were washed three times with 20 mM sucrose, and resuspended in an original volume of pre-heated SCV medium. This timepoint is referred to as 0 h. Samples were subsequently taken every hour and fixed with pre-chilled 70% ethanol for overnight. After centrifugation at 2700 × *g* for 20 min at 25°C, the supernatant was discarded and the cells were resuspended in phosphate-buffered saline (PBS, 1 ml). The sample was centrifuged again at 2700 × *g* for 20 min at 25°C and resuspended in the supernatant (100 μl). The cells were stained with propidium iodide (PI) at a final concentration of 50 μg/ml. After incubation on ice in the dark for 30 min, the samples were analyzed by flow cytometry (Cytek^®^ Amnis^®^, ImageStream^®^X Mk II). A total of 30 000 cells were measured for each sample, and the data were analyzed using the IDEAS data analysis software.

### Enzyme activity assays

The standard reaction mixture for primer synthesis and PADT assays (30 μl) contained 1 μM enzyme, 250 ng of M13mp18 single-stranded DNA (ssDNA) for primer synthesis or 1 μM oligonucleotide dT30dC5 (Supplementary Table S1) for PADT, 10 μM rNTPs (2 μCi [α-^32^P] ATP) in 50 mM 2-morpholinoethanesulphonic acid (MES)–NaOH (pH 6.5), 100 μg/ml bovine serum albumin (BSA), and 10 mM MnCl_2_. The samples were incubated for 30 min at 75°C or 55°C. The reaction mixture (20 μl) for terminal transfer assays contained 0.8 μM enzyme, 100 μM rNTPs, and 4 nM ^32^P-labeled oligonucleotide IC-D25 (Supplementary Table S1) in 50 mM MES–NaOH (pH 6.5), 100 μg/ml BSA, and 10 mM MnCl_2_. The samples were incubated for 15 min at 75°C or 55°C. The standard reaction mixture for microhomology-based annealing assays (20 μl) contained 0.8 μM enzyme, 0.5 μM each of dT32dC3 and dT32dG3 (Supplementary Table S1), 0.5 μCi [α-^32^P] ATP in 50 mM MES–NaOH (pH 6.5), 100 μg/ml BSA, and 10 mM MnCl_2_. The samples were incubated for 30 min at 55°C. The reactions were terminated by the addition of sodium dodecyl sulfate (SDS) and proteinase K to final concentrations of 1% and 1 mg/ml, respectively. After 1 h at 55°C, an equal volume of stop buffer (98% formamide, 0.025% xylene cyanol, 0.025% bromophenol blue, and 10 mM ethylenediaminetetraacetic acid) was added. Samples were boiled for 5 min, quickly cooled on ice, and loaded onto a 20% denaturing polyacrylamide gel (19:1) containing 8 M urea. After electrophoresis in 1×Tris-borate-EDTA (TBE) buffer, the gel was exposed to X-ray film.

### Survival rate determination

*Saccharolobus islandicus* cells were grown to an OD_600_ of ∼0.3 with shaking at 75°C in SCV, SCVyU, or ACV medium. 4-Nitroquinoline-1-oxide (NQO) or methyl methanesulfonate (MMS) was added to a final concentration of 2 μM or 2 mM, respectively. Samples were taken at indicated timepoints, and washed twice with and resuspended in pre-heated SCV medium. The samples were then serially diluted, and the dilutions were plated on SCV, SCVyU, or ACV plates. The plates were incubated at 75°C for 7–10 days for colony counting. The survival rate was obtained by dividing the number of colonies from the treated sample by that from the untreated control sample.

### Mutation analysis

*Saccharolobus islandicus* cells were grown to an OD_600_ of ∼0.3 with shaking at 75°C in SCV, SCVyU, or ACV medium and treated for 6 h with 2 μM NQO or 2 mM MMS. A sample (500 μl) of the culture was taken and spread on a plate containing 120 μM 6-methyl purine (6-MP) and 0.5 mM guanosine monophosphate (GMP). The plates were incubated at 75°C for 7–10 days. Approximately 100 colonies were picked for each sample, and the *apt3* gene as well as its promoter region were sequenced by PCR using the primers Sis-apt3-F and Sis-apt3-R (Supplementary Table S1). The apparent MF was calculated by first dividing the number of colonies on the 6-MP/GMP-containing medium by that on the medium lacking the mutagen, and then dividing the derived number by the dilution factor for the control. The mutation spectrum is determined based on sequencing results.

### *In vivo* DSB repair assays

A 40-bp spacer sequence targeting the nonessential gene *riol1* of *S. islandicus* [44] was prepared by annealing oligonucleotides Riol1-spa-F and Riol1-spa-R (Supplementary Table S1) and inserted into the BspMI site of pSe-Rp, yielding plasmid pSe-Rp_DSB. To test the ability of *S. islandicus* primase and its subunits to repair DSB *in vivo*, the arabinose promoter sequence from pSeSD and the *priS*, *priL*, *priX*, or *priSL* sequence, prepared by PCR from the genomic DNA of *S. islandicus* E233S using the corresponding primer pairs (Supplementary Table S1), were cloned into pSe-Rp_DSB between the SalI and NotI sites in such a way that expression of the primase gene was under the control of the arabinose promoter, producing plasmids pSe-Rp_DSB_PriS, pSe-Rp_DSB_PriL, pSe-Rp_DSB_PriX, and pSe-Rp_DSB_PriSL. These plasmids as well as a control plasmid lacking the target spacer were electroporated into strain E233S. The transformed cells were diluted, and the dilutions were spread on ACV plates. After incubation at 75°C for ∼10 days, colonies were counted for the calculation of survival rates, as described above. The region containing the target spacer (5′-CCTATTCTGGTTTACGAAAACATTCTTGTAATGGAATTTATTG) and the PAM sequence (5′-CCT-3′) was sequenced by using primers Riol1-DSB-F and Riol1-DSB-R (Supplementary Table S1) to allow the analysis of mutations.

### Ionizing radiation

*Saccharolobus islandicus* cells were cultured in SCVyU or ACV medium to an OD_600_ of 0.30∼0.40, and subjected to ionizing radiation (IR) at a total dose of 0, 200, 400, or 800 Gy from a ^137^Cs radiation source (MDS NORDION, Canada; model: GC1000; serial number: 469) emitting γ-radiation at a rate of 11.8 Gy/min at the National Institute of Biological Sciences, Beijing. For growth measurement, the irradiated cells were inoculated into SCVyU or ACV medium pre-warmed to 75°C, and cultured at 75°C with shaking. For cell survival measurement, the irradiated samples were diluted in ACV or SCVyU medium, and the dilutions were plated on ACV or SCVyU plates for colony counting. The survival rate was obtained by dividing the number of colonies from the treated sample by that from the untreated control sample.

### Immunoblotting

Exponentially grown *S. islandicus* cells were harvested and resuspended to the same cell density in PBS. An equal aliquot of each sample was loaded onto an SDS polyacrylamide gel. Following electrophoresis, proteins were electrophoretically transferred onto a polyvinylidene fluoride (PVDF) membrane (Bio-Rad). The membrane was incubated first with a rabbit antiserum against a target protein and then with an anti-rabbit antibody conjugated to horseradish peroxidase (HRP) (Promega). The target proteins were detected with the ECL Western Blot Substrate (Thermo Fisher Scientific) and visualized with a Tanon 5200 Multi Chemiluminescent System (Tanon, Shanghai, China). Intensities of the protein bands were determined by using Quantity One Software (version 4.6.2, Bio-Rad).

## Results

### PriL is involved in the DNA damage response of *S. islandicus*

It was reported that an *S. islandicus* primase chimera comprising wild-type PriS and a fusion protein consisting of the PriL-NTD and N-terminally truncated PriX was capable of primer synthesis as efficiently as the wild-type primase heterotrimer, and thus the Fe–S cluster was not required for primer synthesis *in vitro* [38]. To examine the physiological relevance of this observation, we replaced *priL* with a gene encoding the fusion protein, termed PriLn-Xc, and deleted *priX* in the genome of *S. islandicus* E233S (Supplementary Fig. S1A and B). The PriLn-Xc gene was under the control of the native promoter of *priL*. We found that the resulting strain, denoted CHI-ΔX, was viable and grew as well as the parental strain at 75°C, a temperature optimal for the growth of the archaeon (Supplementary Fig. S1C). Interestingly, CHI-ΔX grew better than the parental strain at 90°C although the growth of both strains was reduced at this temperature (Supplementary Fig. S1C). Despite many attempts, we were unable to construct an *S. islandicus* mutant strain lacking PriL-CTD. Therefore, while the Fe–S cluster-containing PriL-CTD is essential for the *in vivo* role of the heterotrimer PriSLX, the Fe–S cluster *per se* is dispensable for the growth of *S. islandicu*s and may serve a structural role.

**Figure 1.**
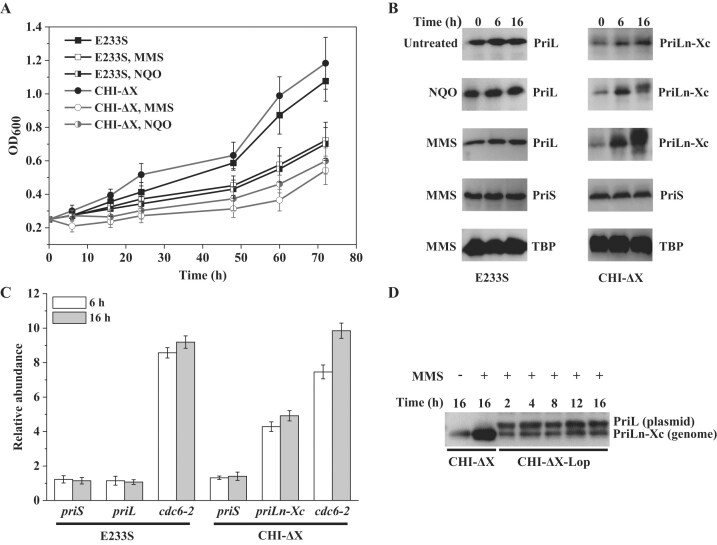
Response of CHI-ΔX and the parental strain to treatment with DNA damaging agents. (**A**) Growth curves of the E233S and CHI-ΔX following DNA damage treatment. Strain E233S and CHI-ΔX were grown with shaking at 75°C to an OD_600_ of ∼0.25, and 2 μM NQO or 2 mM MMS was added, respectively. During further incubation, the OD_600_ values of the cultures were measured. All data points are an average of three independent measurements. (**B**) Effect of NQO or MMS treatment on the cellular contents of primase subunits. Strain E233S and CHI-ΔX were grown with shaking at 75°C to an OD_600_ of ∼0.25, and treated with 2 μm NQO or 2 mM MMS. Samples were taken at indicated times, centrifuged, and resuspended in 1× PBS to the same OD_600_. An equal aliquot of each sample was subjected to sodium dodecyl sulfate–polyacrylamide gel electrophoresis. PriL and PriLn-Xc were detected by immunoblotting with an antibody against PriL, and PriS was detected with an antibody against PriS. *Saccharolobus islandicus* TATA box binding protein (TBP) was used as a loading control and detected with an antibody against TBP. (**C**) Effect of NQO or MMS treatment on the cellular levels of transcripts encoding primase subunits. Strain E233S and CHI-ΔX were grown with shaking at 75°C to an OD_600_ of ∼0.25, and treated with 2 μM NQO or 2 mM MMS. Samples were taken at indicated times, centrifuged, and resuspended in Trizol. RNA was extracted and reverse-transcribed into complementary DNA (cDNA). The transcripts of *priS*, *priL*, *priLn-Xc*, and *cdc6-2* as well as 16S ribosomal RNA were quantified by qPCR. The *cdc6-2* gene, which encodes a master regulator in the network of DNA damage response in *S. islandicus* [51], is used as a positive control. All data points are an average of three independent measurements. (**D**) Response of the cellular PriLn-Xc level to MMS treatment in the presence of plasmid-encoded PriL. CHI-ΔX containing a plasmid encoding PriL under the control of an arabinose promoter was grown in ACV medium in the presence of arabinose and, when the OD_600_ of the culture reached ∼0.3, treated with 2 mM MMS. Samples were taken at indicated timepoints and processed as above for the quantification of PriL and PriLn-Xc by immunoblotting.

To learn more about the physiology of CHI-ΔX, we compared the mutant strain with the parental strain in their response to treatment with a DNA-damaging agent. Both strains were grown to the early exponential phase, and MMS or NQO was added to 2 mM or 2 μM, respectively, a concentration at which ∼80% killing was observed for the parental strain. As shown in Fig. 1A, CHI-ΔX was more sensitive to either MMS or NQO than the parental strain. The cellular level of the fusion protein increased by ∼4.8- or ∼2.6-fold, respectively, following the MMS or NQO treatment (Fig. 1B). Since the cells showed similar patterns of response to the two DNA-damaging agents, we carried out the experiments below on the MMS-treated cells. The increase in PriLn-Xc in the MMS-treated CHI-ΔX appeared to have resulted from the upregulation of the transcription of the gene encoding the protein, as revealed by quantitative PCR (qPCR) (Fig. 1C). No significant changes in the cellular concentration of either PriL or PriS were detected in the parental strain (Fig. 1B and C). Intriguingly, when plasmid-encoded PriL was produced in CHI-ΔX (CHI-ΔX-Lop), the upregulation of the fusion protein in response to the MMS treatment no longer occurred (Fig. 1D). We inferred from these results that PriL was involved in the DNA damage response of *S. islandicus*, and this primase subunit was subjected to regulation involving the PriL-CTD.

**Figure 2.**
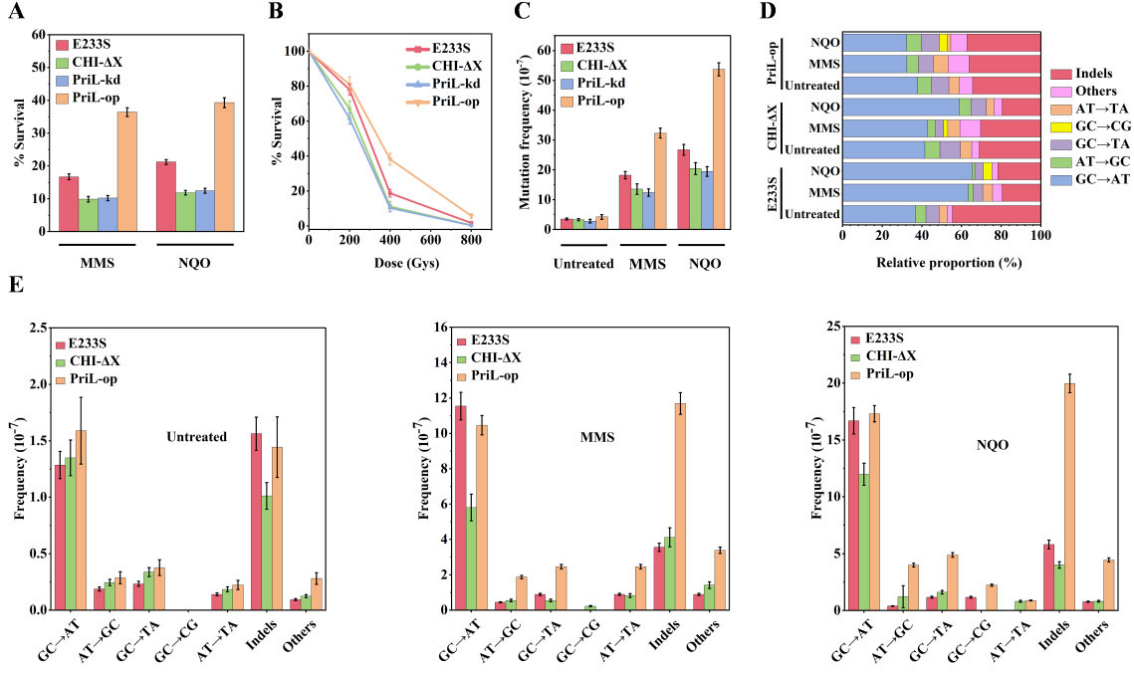
Effect of MMS, NQO, and IR on the survival and mutation of PriL-kd, PriL-op, CHI-ΔX, and the parental strain. (**A**) Effect of MMS or NQO treatment on the survival of PriL-kd, PriL-op, CHI-ΔX, and E233S. Cells were grown with shaking at 75°C to an OD_600_ of ∼0.3. After the addition of 2 μM NQO or 2 mM MMS, samples were taken at intervals, serially diluted, and plated on plates for colony counting. The survival rates were obtained by dividing the number of colonies from the treated sample by that from the untreated control sample. Each number represents an average of three independent measurements. (**B**) Effect of IR on the survival of PriL-kd, PriL-op, CHI-ΔX, and E233S. Cells were grown with shaking at 75°C to an OD_600_ of 0.30∼0.40, and subjected to γ-radiation at indicated doses. The cells were taken at intervals, serially diluted, and plated on plates for colony counting. The survival rates were obtained by dividing the number of colonies from the treated sample by that from the untreated control sample. Each number represents an average of three independent measurements. (**C**) Effect of MMS or NQO treatment on the frequency of formation of 6-MP-resistant mutants in PriL-kd, PriL-op, CHI-ΔX, and E233S. Exponentially grown cells were treated with 2 μM NQO or 2 mM MMS, and samples were spread on plates containing 120 mM 6-MP and 0.5 mM GMP. The MF was calculated by first dividing the number of colonies on the 6-MP/GMP-containing medium by that on the medium lacking the mutagen, and then dividing the derived number by the dilution factor. (**D**) Spectra of mutation in the *apt3* gene from the 6-MP-resistant cells of PriL-op, CHI-ΔX, and E233S. Colonies on the 6-MP/GMP plates, obtained in experiments shown in Fig. 2C, were picked, and the *apt3* gene as well as its promoter region were sequenced. The sequencing results of 100 6-MP-resistant colonies for each strain were analyzed. Types of mutation, including BPSs and indels, are shown. Others: Other types of mutation. (**E**) Effect of MMS or NQO treatment on the frequencies of different types of mutation in PriL-op, CHI-ΔX, and E233S. The MF of a given type of mutation is calculated by multiplying the frequency of total mutations (Fig. 2C) by the fraction of this type of mutation in the total mutations (Fig. 2D).

To further explore the role of PriL in DNA damage response by *S. islandicus*, we constructed a PriL-kd and a PriL-op strains (Supplementary Fig. S2), the expression of their PriL is regulated by the arabinose promoter. The two strains, together with CHI-ΔX and the parental strain, were tested for their rates of survival, as measured by colony counting, following treatment with MMS or NQO (Fig. 2A). The cellular levels of PriL in PriL-kd and PriL-op were ∼0.71- and ∼3.14-fold, respectively, as high as that in the parental strain (Supplementary Fig. S2). After the MMS treatment, the rate of survival for CHI-ΔX (∼9.88%) was substantially lower than that for the parental strain (∼16.66%). PriL-kd was also more susceptible to the treatment (∼10.20%) than the parental strain, as expected. In comparison, the survival rate of PriL-op (∼36.40%) was more than twice as high as that of the parental strain (Fig. 2A and Supplementary Table S2). A similar survival pattern was also observed following the treatment of the four strains with NQO, with the survival rates of approximately 21.2%, 11.83%, 12.45%, and 39.3% for the parental strain, CHI-ΔX, PriL-kd, and PriL-op, respectively (Fig. 2A and Supplementary Table S2). We conclude that an increase in the cellular content of wild-type PriL allows the cells to better survive DNA damage induced by MMS and NQO.

MMS has been shown to generate DSBs in hyperthermophiles [45], and 4-NQO is also potentially capable of inducing indirectly DNA DSBs [46–48]. We therefore speculated from the above experiments that PriL might be involved in the resistance of *S. islandicus* to DSBs. To test this possibility, we examined the ability of the cells to survive IR, a standard method for generating DSBs in the cell [49, 50]. PriL-kd, PriL-op, CHI-ΔX, and E233S were grown to the exponential phase, and the culture of each strain was subjected to ^137^Cs γ-radiation at a dosage of 0, 200, 400, or 800 Gy. The survival rates of the irradiated cells were then determined by plate counting. As shown in Fig. 2B, the rates of survival of the parental strain E233S were 78.00%, 18.75%, and 1.75%, respectively, at 200, 400, and 800 Gy. This level of γ-rays tolerance is similar to that shown previously for *S. solfataricus* [50]. In comparison, the survival rate of PriL-op was higher, and those of PriL-kd and CHI-ΔX were lower, than that of the parental strain at each of the γ-ray doses used. The pronounced difference between the strains in sensitivity to killing by radiation was clearly shown at 400 Gy. At this dose, the survival rate of PriL-op (38.25%) was over twice as high as that of E233S (18.75%) and 3- to 4-fold as high as those of CHI-ΔX (11.18%) and PriL-kd (10.27%). After radiation at 800 Gy, the parental strain was barely able to survive (1.75%), and CHI-ΔX and PriL-kd were nearly all killed with survival rates of 0.45% and 0.35%, respectively. On the other hand, PriL-op exhibited a survival rate of 5.75%. Indeed following exposure to 800 Gy, PriL-op showed only a moderate delay in growth whereas PriL-kd could hardly grow (Supplementary Fig. S3). Taken together, these results are consistent with a role for PriL in the ability of *S. islandicus* to survive the stress of DSBs.

### PriL enhances error-prone DNA repair in *S. islandicus*

We then asked if the observed rates of the survival of the tested strains following DNA damage treatment correlated with the MFs in the cells. To test this possibility, we employed a mutation assay involving the use of the purine analog 6-MP. Since incorporation of 6-MP into DNA, as facilitated by adenine phosphoribosyltransferase encoded by *apt3*, kills *S. islandicus* cells, only APT3-defective mutants can grow on a 6-MP plate [52]. As shown in Fig. 2C and Supplementary Table S3, the parental strain, CHI-ΔX, PriL-kd, and PriL-op showed similar apparent MFs in spontaneous mutation (2.81 × 10^–7^∼4.20 × 10^–7^). Following MMS or NQO treatment, the MF of the parental strain increased to 1.82 × 10^–6^ or 2.67 × 10^–6^, respectively, whereas those of CHI-ΔX and PriL-kd also increased, but to a slightly lesser extent (1.36 × 10^–6^ or 2.04 × 10^–6^ for CHI-ΔX and 1.24 × 10^–6^ or 1.94 × 10^–6^ for PriL-kd). Notably, a drastic increase in MF (3.23 × 10^–6^ or 5.37 × 10^–6^) after MMS or NQO treatment was observed in PriL-op (Supplementary Table S3). Therefore, the MF in cells exposed to DNA damage treatment appears to correlate positively with the cellular level of wild-type PriL.

We subsequently looked into the spectrum of the mutation in the parental strain, CHI-ΔX, and PriL-op by picking approximately one hundred 6-MP-resistant colonies for each strain, and sequencing a region spanning *apt3* and its putative promoter (Fig. 2D). Nearly all of the 300 colonies contained at least one mutation located mostly (93.30%) in the coding region and occasionally (6.70%) in the promoter region of *apt3*. Small insertions or deletions (1–2 bp indels) and base pair substitutions (BPSs) were predominant among the various types of mutation in all three strains (Fig. 2D). Indels accounted for 44.65%, 31.13%, and 34.38%, respectively, of the detected mutations in the parental strain, CHI-ΔX, and PriL-op (Supplementary Table S4). Very few indels were longer than 2 bps, and these included insertion sequence (IS)-mediated insertions and large deletions. BPSs showed a strong bias for GC→AT transition, which accounted for 66.31%, 60.26%, and 59.22%, respectively, of the total detected BPSs in the parental strain, CHI-ΔX, and PriL-op. Other BPSs included base substitutions, such as CC→TT, AT→CG, and AT→TA. The observed mutation spectrum resembles that reported previously for a different *S. islandicus* strain using the same assay [52].

The frequencies of indels and BPSs changed significantly but differently, resulting in changes in mutation spectrum, in the three strains following treatment with a DNA damaging agent. The fraction of indels in the total detected mutations decreased by 2.29-fold but that of GC→AT increased by 1.73-fold in the parental strain treated with MMS, as compared with those in the untreated control (Fig. 2D and Supplementary Table S4). Therefore, in the MMS-treated parental strain, GC→AT transition (63.41%) far outnumbered indels (19.51%). In comparison, the frequencies of GC→AT transition and indels increased by 4.30- and 4.07-fold (Fig. 2E and Supplementary Table S5), respectively, and the proportion of GC→AT (42.86%) was slightly higher than that of indels (30.41%) in the MMS-treated CHI-ΔX (Fig. 2D and Supplementary Table S4). Notably, the frequencies of both GC→AT transition and indels increased drastically by 6.56- and 8.09-fold, respectively (Fig. 2E and Supplementary Table S5), and indels (36.19%) were more than GC→AT transitions (32.38%) in the MMS-treated PriL-op (Fig. 2E and Supplementary Table S5). Similar observations were also made on the changes in mutation profile in these strains following NQO treatment (Supplementary Tables S4 and S5). Based on the effect of DNA damage on the rate and spectrum of mutation in these strains, we conclude that wild-type PriL is involved in error-prone DNA repair, resulting preferentially in the occurrence of small indels.

### *Saccharolobus islandicus* PriSL repairs DSBs

**Figure 3.**
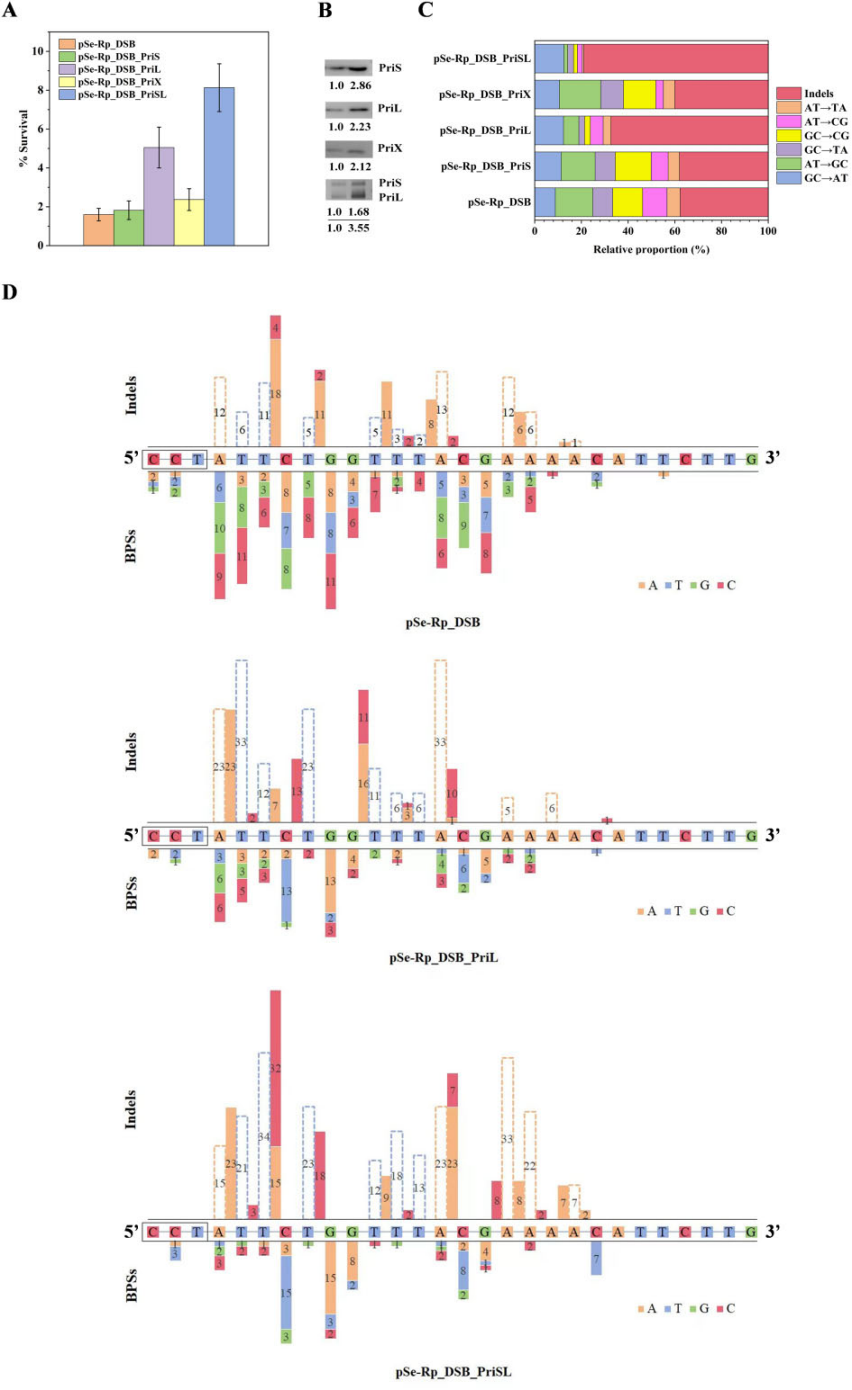
Error-prone repair of DSBs by PriL *in vivo*. (**A**) Effect of plasmid-encoded primase subunits on the resistance of *S. islandicus* to endogenously generated DSBs. E233S cells were transformed by electroporation with plasmid pSe-Rp_DSB_PriS, pSe-Rp_DSB_PriL, pSe-Rp_DSB_PriX, or pSe-Rp_DSB_PriSL, and the transformed cells were diluted and spread on ACV plates for colony counting. The fraction of survival was calculated by dividing the number of colonies from the cells transformed with the above plasmids by that from the control cells transformed with a control plasmid lacking the protospacer. Each number represents of an average of three independent measurements. (**B**) Cellular levels of genome- and plasmid-encoded primase subunits. Exponentially grown cells of strain E233S containing pSe-Rp_DSB_PriS, pSe-Rp_DSB_PriL, pSe-Rp_DSB_PriX, and pSe-Rp_DSB_PriSL were subjected to immunoblotting. The ratio of plasmid-encoded copy (upper band) to genome-encoded copy (lower band) of each tested primase subunit is indicated. (**C**) Spectrum of mutations in the 40-bp target region in surviving cells following the generation of DSBs by a CRISPR–Cas system. Colonies were picked from the plates obtained from the experiments shown in Fig. 3A. The 40-bp spacer region was sequenced. Mutated sites of the 40-bp spacer were identified and classified into different types based on the nature of the mutations. (**D**) Hotspots of DSB-induced mutation in the 40-bp spacer and the PAM sequence (5′-CCT-3′) of strains transformed with pSe-Rp_DSB, pSe-Rp_DSB_PriL, and pSe-Rp_DSB_PriSL. Base insertions (colored bar) and deletions (hollow bar) are indicated above the target sequence. BPSs are shown below the target sequence. Only the part of the target sequence where mutations were detected is shown. Numbers represent the number of occurrences.

Since indels are often generated during DSB repair, we then looked directly into the involvement of PriL in DSB repair and characterized the repair products by using an assay in which a site-specific DSB was generated *in vivo*. We constructed a type-IA CRISPR-based gene editing plasmid (pSe-Rp_DSB) targeting a 40-bp sequence in the nonessential *riol1* gene [44]. When the plasmid was introduced into the parental strain, 98.40% killing was observed (Fig. 3A), suggesting the successful generation of DSBs, as expected from previous studies [53, 54]. The gene encoding PriL, PriS, PriX, or PriSL was then cloned into pSe-Rp_DSB and placed under the control of the inducible arabinose promoter. The parental strain was transformed with these plasmids. Upon induction, the cellular contents of these primase subunits increased to similar extents and were all over twice as high in the overproducing strains as in the untransformed control strain (Fig. 3B). Of the three primase subunits, PriL most drastically increased the rate of cell survival when overproduced alone (3.16-fold) (Fig. 3A and Supplementary Table S6). Overproduction of PriS showed little effect on cell survival (1.14-fold), whereas overproduction of PriX only moderately enhanced the survival of the cells (1.48-fold). When PriL and PriS were co-overproduced, the rate of cell survival increased further (5.08-fold), as compared with that in the PriL overproducer, probably due to the higher PriL content in the PriSL overproducer than in the PriL overproducer (1.59-fold) (Fig. 3B). PriS, PriL, and PriX exist at a molar ratio of ∼13:2:1 in the exponentially growing *S. islandicus* cells [37]. The catalytic subunit PriS is in large molar excess over either PriL or PriX. The failure of PriX to substantially enhance the cell survival appears to preclude a major role for this subunit in DNA repair. On the other hand, PriL is likely a limiting factor in the PriL-mediated DNA damage response in this organism.

To understand how cells survive the genome cleavage by the CRISPR–Cas system, we picked surviving colonies on the plates and sequenced the 40-bp protospacer and the PAM sequence (CCT). Approximately 98% of the tested colonies contained at least one mutation in the target sequence. Most of the remaining colonies had a mutation in the PAM sequence. Nearly all mutations were found within the upstream half of the protospacer as well as the PAM region. Bias for this part of the target sequence is consistent with the cleavage pattern of the type-IA CRISPR system [55].

Indels accounted for 37.7% of the mutations in the parental strain, and 67.4% and 79.0%, respectively, in the PriL and PriSL overproducers (Fig. 3C and Supplementary Table S7). There were significantly more 1- to 2-bp indels (∼85%) than >2-bp indels (∼15%). Interestingly, while insertions occurred exclusively following A or C, deletions were only found at A or T, despite the randomness of the target sequence (Fig. 3D). Strikingly, in a more careful analysis, we found that insertion preferentially resulted in the formation of CC (insertion of a C following C), GGC (insertion of a C following GG), CGG (insertion of a C before GG), or CCG (insertion of a C in the middle of CG) in the DNA strand. This preference was far more evident in the overproducers of PriL and PriSL (14.23% and 17.10%, respectively, of the total indels), as compared with that in parental strain (5.68%) (Fig. 3D). We have previously shown that PriSL and PriSLX are able to catalyze template-dependent PADT [17, 37]. In the PADT reaction, the primase promotes annealing between the 3′-end of a strand with a template and extends the annealed strand at the 3′-end. The alternative possibility that PADT products result from snap-back synthesis was ruled out. The primase-promoted annealing between DNA strands sharing microhomology is a key step in PADT. PriSL was shown to be able to promote annealing between strands containing as short as 2–4 contiguous GC pairs [17]. Therefore, the pattern of insertion observed in this study provides strong support for a role of the primase in DSB repair.

**Figure 4.**
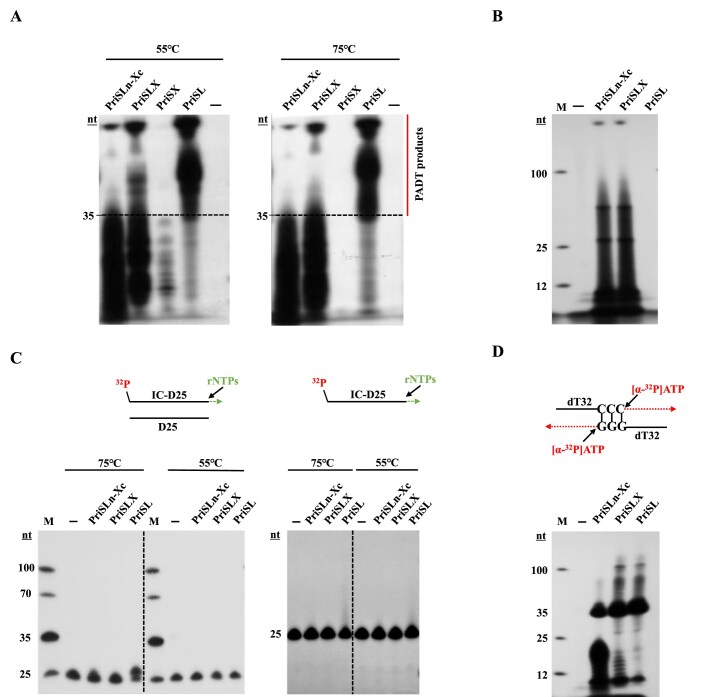
Comparison of the activities of PriSLn-Xc, PriSLX, and PriSL. (**A**) PADT. Reactions were performed by incubating PriSLn-Xc, PriSLX, or PriSL (1 μM) with oligonucleotide dT30dC5 (1 μM) and 10 μM rNTPs (2 μCi [α-^32^P] ATP) at 55°C (left panel) and 75°C (right panel) in the standard assay mixture. (**B**) Primer synthesis. Reactions were performed by incubating PriSLn-Xc, PriSLX, or PriSL (1 μM) with M13mp18 ssDNA (250 ng) in the presence of 10 μM rNTPs (2 μCi [α-^32^P] ATP) at 75°C in the standard assay mixture. (**C**) Terminal transfer. Reactions were performed by incubating PriSLn-Xc, PriSLX, or PriSL (0.8 μM) with a duplex DNA fragment (4 nM, left), prepared by annealing a ^32^P-labeled 25-bp oligonucleotide (IC-D25) with an unlabled complementary 25-nt oligonucleotide (D25), or a ^32^P-labeled IC-D25 (4 nM, right) in the presence of rNTPs (100 μM) at 75°C or 55°C in the standard assay mixture. Sketches of the labeled-templates are shown on top of the gel images. (**D**) Microhomology-based annealing. Microhomology-based annealing was carried out by incubating PriSLn-Xc, PriSLX, or PriSL (0.8 μM) with both dT32dC3 and dT32dG3 (0.5 μM each) in the presence of [α-^32^P] ATP (0.5 μCi) at 55°C. Sketches of the DNA templates are shown on top of the gel images. All of the above reactions were stopped by the addition of SDS (1%) and protease K (1 mg/ml). The products were phenol extracted and ethanol precipitated, and subjected to electrophoresis in denaturing polyacrylamide gel electrophoresis (20% polyacrylamide). The gel was exposed to X-ray film.

To determine if PriSL and PriSLX might differ in function at the physiological temperature of *S. islandicus*, we looked at the temperature dependence of various activities of the two complexes (Fig. 4A and B). PriSL was far less active than PriSLX in primer synthesis at 55°C, judging by the amount of products shorter than 35 nt (the size of the oligonucleotide template), but was nearly as active as the latter in PADT, as revealed by the amount of products longer than 35 nt (Fig. 4A). However, when temperature was raised to 75°C, PriSL was barely active whereas PriSLX remained robustly active in primer synthesis (Fig. 4B). On the other hand, PriSL was substantially more active than PriSLX in PADT (Fig. 4A). This is consistent with the observation that PriSL was more active than PriSLX at 75°C in terminal transfer (Fig. 4C), which would permit the initiation of PADT. Therefore, PriSL fitted better than PriSLX for PADT at the optimal growth temperature of the organism. We also found that PriSLn-Xc, an equal molar mixture of PriS and PriLn-Xc, resembled PriSLX in primer synthesis as reported previously [38], but synthesized very little PADT products (Fig. 4A) apparently because of its failure to promote microhomology-mediated strand annealing (Fig. 4D). Taken together, our data suggest that the primase heterodimer PriSL serves a pivotal role in DSB repair in *S. isalnadicus*.

### PriL is cell-cycle regulated

**Figure 5.**
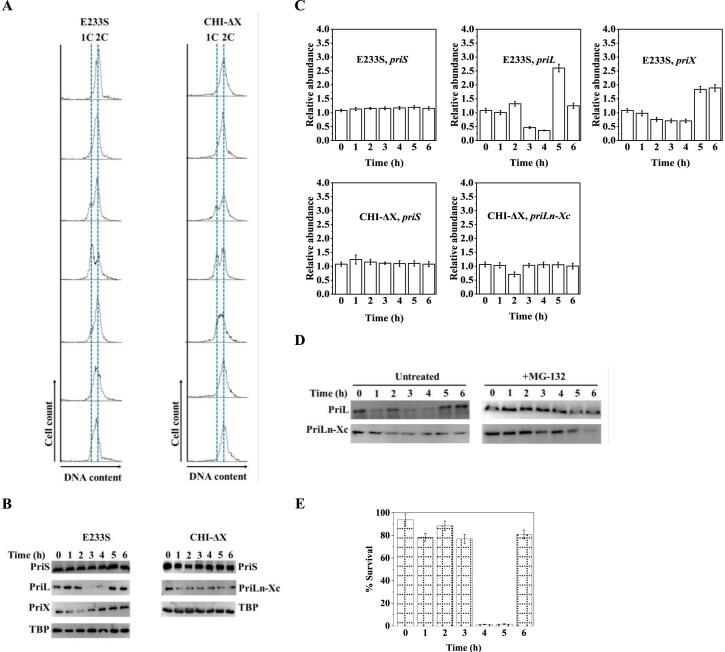
Cell-cycle dependence of the expression of *priL* and the response of *S. islandicus* to DNA damage treatment. (**A**) Cell-cycle analysis. Strain E233S and CHI-ΔX were grown at 75°C with shaking to OD_600_ of ∼0.2, and the growth of the cells was arrested in G2 by treatment with acetic acid (6 mM). Following washes, cells were resuspended in pre-warmed fresh medium to allow the growth of the cells to reoccur, and samples were taken at different timepoints (0, 1, 2, 3, 4, 5, and 6 h from top) during the subsequent incubation. The samples were fixed with ethanol, washed with 1× PBS, and stained with PI (50 μg/ml). The cells were then analyzed by flow cytometry.(**B**) The cellular contents of primase subunits during the cell cycle. Cells were synchronized as above. After the release of the cells into fresh medium, samples were taken at intervals, and PriL, PriLn-Xc, PriS, and PriX were quantified by immunoblotting with antibodies against PriL (for PriL and PriLn-Xc), PriS (for PriS), and PriX (for PriX). (**C**) The cellular levels of transcripts encoding *priS*, *priL*, *priX*, and *priLn-Xc* during the cell cycle. Cells were synchronized as shown in Fig 5A. The synchronized cells were grown with shaking at 75°C. Samples were taken at intervals, and RNA was extracted and reverse-transcribed into cDNA. Transcripts of *priS*, *priL*, *priX*, and *priLn-Xc* were quantified by qPCR. All data points are an average of three independent measurements. (**D**) Effect of MG-132 on the cell-cycle progression of PriL. Cells were synchronized as above, and released into fresh medium containing MG-132 (25 μM). Samples were taken at intervals for immunoblotting with antibodies against PriL. (**E**) Effect of NQO treatment on the survival of *S. islandicus* synchronized in different phases of cell cycle. Strain E233S was grown at 75°C with shaking to an OD_600_ of ∼0.2, synchronized by treatment with 6 mM acetic acid, and released into fresh medium. Samples were taken at 1 h intervals, treated for 15 min with 2 μM NQO, diluted, and plated for colony counting. The survival rates were obtained by dividing the number of colonies from the NQO-treated sample by that from the untreated control sample. Each number represents an average of three independent measurements.

Since the cellular level of PriL appeared to be critical in the DNA damage response of *S. islandicus*, we sought to determine if it was regulated during a cell cycle. Both the parental strain and CHI-ΔX were synchronized to G2 phase through the acetic acid treatment, and the cell cycle of each strain was monitored by flow cytometry. No significant differences were detected between the two strains in cell-cycle progression, suggesting that fusion of PriL-NTD and N-terminally truncated PriX did not interfere with the cell cycle (Fig. 5A), in agreement with the lack of differences in growth phenotype between the two strains under the optimal growth conditions. We then determined the expression of genes encoding the primase subunits at different timepoints during the cell cycle. In the parental strain, PriS existed at a relatively constant level throughout the cell cycle, whereas PriX showed moderate variation in cellular concentration with the lowest level at 2 h (Fig. 5B). Surprisingly, PriL became nearly undetectable at 3∼4 h, but the protein was readily detected and existed at a nearly unchanged level at other timepoints (Fig. 5B). By comparison, neither PriS nor PriLn-Xc varied significantly in cellular concentration throughout the cell cycle in CHI-ΔX (Fig. 5B). At the transcriptional level, *priS* appeared to be constitutively transcribed in the parental strain (Fig. 5C). However, the transcription of both *priL* and *priX* was regulated in a cell-cycle-dependent manner. Notably, few transcripts of *priL* were detected at 3∼4 h, the same timepoints when cellular PriL content was at the lowest level (Fig. 5B and C). Interestingly, both *priL* and *priX* transcripts were most abundant at 5 h and, for *priX*, even at 6 h (Fig. 5C). Since the cells were in late S/early G2 at 3∼4 h, our results suggest that PriL was rapidly lost after genomic DNA replication. We speculate that the loss of PriL was due to the pause of the transcription of its coding gene as well as the degradation of the existing protein. Resumption of the transcription of *priL* occurred in G2 (after 5 h). On the other hand, transcription of *priS* and *priLn-Xc* varied only slightly during the cell cycle in CHI-ΔX (Fig. 5C). Since *priL* and *priLn-Xc* share the *priL-NTD* sequence as well as the sequence upstream of the genes, PriL-CTD was probably involved in the cell-cycle regulation of PriL.

To further verify the cell-cycle dependence of the cellular content of PriL, we blocked cell division by adding MG-132, an inhibitor of the 20S proteasome whose activity has been shown to be required for *S. acidocaldarius* cell division [56], to the synchronized cells and releasing the treated cells into fresh medium. As revealed by immunoblotting, no significant decrease in the cellular level of PriL occurred and the protein content remained constant during the subsequent incubation (Fig. 5D). These results indicate that the cellular content and function of PriL are strictly regulated by cell cycle in *S. islandicus*.

In view of the cell-cycle dependence of the cellular level of PriL and the proposed role of PriL in DNA damage response by *S. islandicus*, we then asked if the survival rate of the archaeon following DNA damage treatment would vary during the cell cycle. Strain E233S was synchronized and treated with NQO in different phases of the cell cycle, and the rates of survival were determined. As shown in Fig. 5E, the strain was most sensitive to the NQO treatment at 4∼5 h post-synchronization with the rate of survival (Supplementary Table S8) dropping to <5% from 75%∼95% at other timepoints. This pattern is in general agreement with the variation of the PriL content during the cell cycle (Fig. 5B). The slight delay in the increase in sensitivity of the cells to the NQO treatment, as compared with the decrease in PriL content, may have resulted from differences between the two assays in experimental manipulation. Therefore, we conclude that PriL mediates DSB repair in a cell-cycle-dependent manner.

## Discussion

Replacement of PriL and PriX with a fusion protein consisting of the PriL-NTD and the C-terminal portion of PriX showed little impact on the growth of *S. islandicus* under optimal conditions but compromised the ability of the organism to survive treatment with the chemical mutagens NQO and MMS and exposure to DSB-generating IR. This observation, as well as the upregulation of the fusion protein in CHI-ΔX, but none of the three primase subunits in the parental strain, in response to the NQO or MMS treatment suggest that a constitutive level of PriL is sufficient for the organism to cope with the amount of the incurred DNA damage. Conceivably, the fusion protein is unable to serve the role of PriL in DNA damage response. Consistent with these findings, the rate of survival increased in the PriL overproducer and decreased in the PriL-kd mutant, as compared with that in the parental strain following the treatment with the DNA damaging agents. On the other hand, overproduction of PriS or PriX had little or only slight effect on the DNA damage response of the cells. Furthermore, we show that the increased cell survival in the presence of an elevated cellular level of PriL following DNA damage treatment results from enhanced error-prone DNA repair. We then provide direct evidence that PriL is involved in the repair of DSBs generated by a CRISPR–Cas system at a specific site in the genome. These results support the notion that *S. islandicus* possesses a novel and efficient DSB repair pathway in which PriL plays a pivotal role.

The PriL-mediated DSB repair is distinctly different from a typical form of either NHEJ or MMEJ, two well-studied DSB repair pathways. In NHEJ, Ku, an essential protein, binds to the ends of DSB and recruits proteins involved in NHEJ repair [57]. NHEJ is facilitated by microhomology often present in single-stranded overhangs at the DNA ends. No proteins homologous to Ku have been identified in *S. islandicus*. However, PriL may serve a role similar to that of Ku in DSB repair through its ability to bind to DNA and interact with PriS and possibly other proteins required for the DSB repair. Indeed, we have found that PriL interacts with Rad50 and NurA, two DNA repair proteins, by using co-immunoprecipitation (unpublished results). However, it is unclear how these interactions are involved in PriL-mediated DSB repair. On the other hand, authentic MMEJ, which does not depend on Ku, is initiated by end resection at the DSB ends by the MRN nuclease in mammalian cells [58]. The resulting single-stranded overhangs anneal with microhomology, often involving only a few base pairs [59]. Polymerase theta-mediated end joining, a special type of MMEJ, is able to repair DNA breaks using as short as 1 bp of homology [60]. DSB repair through MMEJ exists in Archaea, as most clearly shown in the study of CRISPR–Cas systems in *Haloferax volcanii*, but the mechanistic details remain to be explored [61–63]. Whether end resection is involved in PriL-mediated DSB repair remains to be determined. However, the possibility exists that terminal transfer by PriS, which has been shown to be responsible for PADT [17], may allow the generation of single-stranded overhangs at the DSB ends for the initiation of the repair process.

Archaeal PriS, a member of the Prim-Pol superfamily, has been shown to participate, either alone or in complex with the noncatalytic subunit(s), in an array of *in vitro* reactions including TLS and repriming, in addition to primer synthesis and extension [9, 21]. Slight upregulation of the primase subunits was observed in *Sulfolobus* cells following UV radiation [64]. We have previously found, before the identification of PriX, that PriSL from *S. solfataricus* is capable of PADT *in vitro*. PADT involves primase-facilitated annealing of the 3′-end of a DNA strand, with or without the addition of nucleotides through template-independent terminal nucleotidyl transfer by the enzyme, to another strand, requiring only minimum sequence homology (preferably 2–4 contiguous GC pairs), and strand extension at the annealed 3′-end [17]. PriX, PriL, and PriS exist at an ∼1:2:13 molar stoichiometry [37], and thus PriS is in large molar excess over either noncatalytic subunit *in vivo*. Given the known interactions between the three subunits, the two catalytically active complexes PriSL and PriSLX would presumably exist, possibly undergoing dynamic interchanges, in the cell. Our results suggest that the two complexes serve distinctly different roles *in vivo*. Primer synthesis is catalyzed primarily by PriSLX, whereas PADT is performed by PriSL at 75°C, the optimal growth temperature of the organism. PriX, an essential noncatalytic subunit of the *S. islandicus* responsible for the initiation step of primer synthesis [37, 38, 65], is not required for the DSB repair activity of the primase in agreement with the finding that PADT may occur in the absence of primer synthesis. The remarkable ability of PriSL to facilitate annealing between strands sharing microhomology is essential for DSB repair through NHEJ, as it is for PADT. It is worth noting that PriSLn-Xc is incapable of PADT, consistent with the compromised ability of the mutant strain to survive DSBs. Since PriS does not bind DNA, binding of the primase to template DNA depends on the interaction between PriL and the DNA. However, the role of PriL in PriSL-facilitated strand annealing is not limited to endowing the primase with the DNA binding ability since PriLn-Xc which bound DNA as well as PriL failed to promote strand annealing when in complex with PriS (Supplementary Fig. S4).

**Figure 6.**
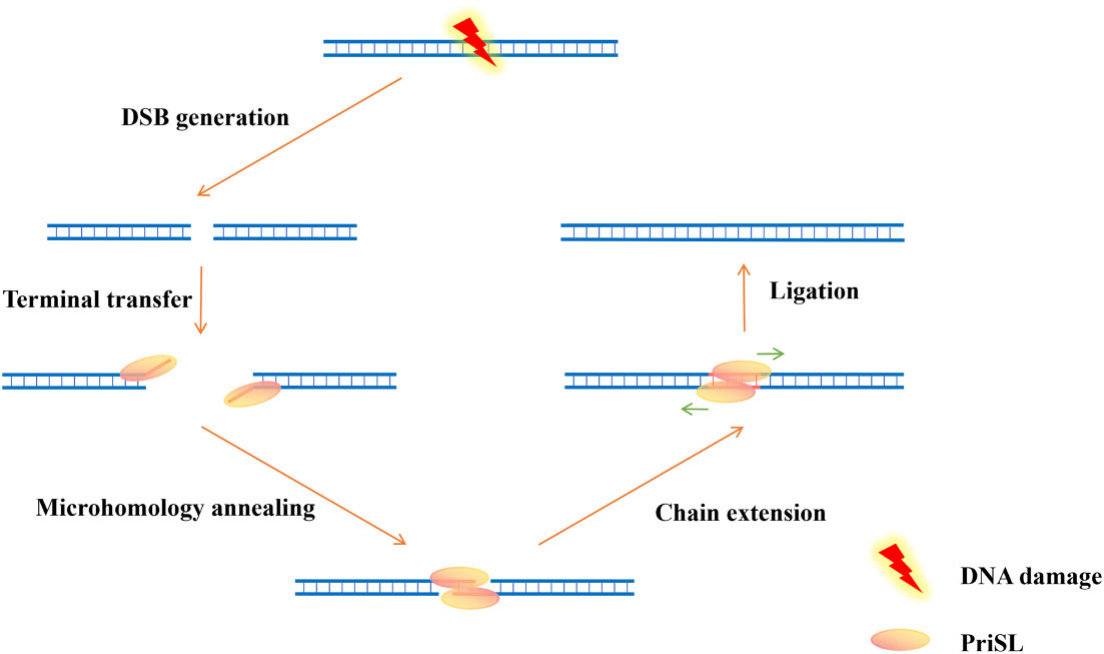
Model of PriSL-mediated DSB repair. When a DNA DSB occurs, PriSL binds directly or is recruited to the site of the DSB. PriS in the DNA-bound PriSL heterodimer is positioned proximal to the 3′-end of a DNA strand on one side of the DSB. Facilitated by PriL, the 3′-end, with or without the addition of nucleotides by terminal transfer reaction, searches for and anneals to a sequence with microhomology, at the 3′-end of a DNA strand similarly bound by PriSL, on the other side of the break point. Once the broken ends are bridged, chain extension by PriSL occurs, followed by polishing through the activities of nucleases, DNA Pol and Lig.

Based on this study and the previous results, we propose the following model for the mechanism underlying DSB repair through MMEJ involving PriSL (Fig. 6). PriSL binds directly or is recruited through interaction with an unidentified DSB recognition protein or complex to the site of a DSB. PriS in the DNA-bound heterodimer is positioned proximal to the 3′-end of a DNA strand on one side of the DSB. This 3′-end, which may be extended by template-independent terminal transfer, then search for and anneal to a sequence with microhomology at the 3′-end of another DNA strand, which is similarly bound by PriSL, on the other side of the break point. Once the broken ends are bridged, chain extension from the annealed 3′-ends by PriSL occurs. It was previously reported that rNTPs are preferred substrates over dNTPs for PADT [17], and a high rNTP/dNTP ratio exists in *Saccharolobus* [66]. Therefore, the products of the PriSL-catalyzed synthesis are subjected to polishing, including the replacement of the RNA with DNA, through the activities of nucleases, DNA Pol and Lig.

Many bacteria but only very few archaeal species, such as *M. paludicola*, possess a simplified NHEJ complex consisting of Ku and LigD, which form an NHEJ complex capable of repairing DSBs generated in the stationary or sporulation stages of the growth cycle [9, 31, 67, 68]. Requiring no Ku or LigD, the PriSL-mediated DSB repair therefore represents a DSB repair pathway with unique enzymology, which we propose to name as pp-MMEJ for Prim-Pol-promoted MMEJ. Since both PriS and PriL homologs are highly conserved, this pathway may be widely employed in Archaea.

The pp-MMEJ pathway appears highly efficient since no significant upregulation of either PriS or PriL was observed in cells treated with the DNA damaging agents under our experimental conditions. Intriguingly, the expression of PriLn-Xc, whose encoding gene was under the control of the same promoter as that of *priL*, was upregulated in response to DNA damage. However, the DNA damage-induced overproduction of PriLn-Xc no longer occurred in the presence of plasmid-encoded wild-type PriL. Therefore, although regulation of the *priL* expression remains to be understood, it must involve a role for PriL.

Interestingly, the cellular level of PriL displayed strong cell-cycle dependence. The cellular content of PriL dropped rapidly to a level hardly detectable around the S/G2 transition. In comparison, the cellular level of PriS was relatively constant and that of PriX varied moderately throughout the cell cycle. Similar cell-cycle patterns were also observed for the transcription of genes encoding the three subunits. However, no significant variation in *priL* expression was found in an earlier genome-wide transcriptomic analysis of the cell cycle of *S.acidocaldarius* [69]. The discrepancy may result from differences in experimental design. It appears that the cell cycle had yet to proceed to completion in the previous study, and the last sample for cell-cycle analysis was taken <3 h after the release of the cells and thus likely before the significant decrease in cellular PriL. We speculate that the cellular PriL was depleted once DNA replication was completed, and the PriSL-mediated DSB repair became nonfunctional during this stage in the cell cycle. Indeed, it was around this stage when synchronized *S. islandicus* cells showed the highest sensitivity to treatment with NQO. Therefore, the PriSL-mediated DSB repair pathway differs from the known NHEJ pathways which function throughout the entire cell cycle. To the best of our knowledge, it has never been shown that the cellular content of a PriL homolog could drop to a negligible level during the cell cycle in any other archaea or eukaryotes. While the observed cell-cycle dependence of PriL is consistent with the cell-cycle regulation of DNA replication, its significance to the DSB repair strategy of the cell is unclear. In conclusion, PriL, which is less well understood as compared with PriS, appears under multilayered control and plays a far more important regulatory role than previously thought in the cell physiology, especially in the DNA damage response of *S. islandicus*.

## Supplementary Material

gkaf322_Supplemental_Files

## Data Availability

The data underlying this article are available in the article and in its online supplementary material.
