# Supplementary material for: Archaeal replicative primase mediates DNA double-strand break repair
Source: Nucleic Acids Res. 2025 Apr 24;53(8):gkaf322. doi: 10.1093/nar/gkaf322 (PMC12019639; doi:10.1093/nar/gkaf322)
Supplement: gkaf322_Supplemental_Files [file gkaf322_supplemental_files.zip › Supplementary material.docx]

**Supplementary material**

**Table S1. Oligonucleotides used in this study**

| Designation | Sequence (5’-3’) |
| --- | --- |
| Lspa-F | AAAGTGTATAGAAAACATTTTAGGGAAGGAAAAGTTAAGCGAAG |
| Lspa-R | TAGCCTTCGCTTAACTTTTCCTTCCCTAAAATGTTTTCTATACA |
| L_L_-SalI-F | ACGCGTCGACATGGTATTAGACGTTAAGAAGTATCCT |
| LSOE-R | GGCGGAAATTTTCCTTCCACTTTTCCTCTCCTTTCTATCAAGCTC |
| Xc-F | AAAGTGGAAGGAAAATTTCCGCC |
| Xc-R | GAGATAATGAAAGCATTAAAAAATAACTAG |
| LSOE-F | AAAGTGGAAGGAAAATTTCCGCC |
| L_R_-NotI-R | ATAAGAATGCGGCCGCTTTAGCTGTCATCCCGTATCACTAG |
| Xspa-F | AAAGGCTGGACACATAGAGTATGCCGATGGTTTTTCCTCAATCT |
| Xspa-R | TAGCAGATTGAGGAAAAACCATCGGCATACTCTATGTGTCCAGC |
| X_L_-SalI-F | ACGCGTCGACCTCTATTCTTTTCAGTTATCAC |
| XSOE-R | CATAATGGATCTTTTTCATGAAATAATAGATGATTGATACTCAAGG |
| XSOE-F | ATTTCATGAAAAAGATCCATTATG |
| X_R_-NotI-R | ATAAGAATGCGGCCGCCCAGCTTTCATTCCTTCTTTTG |
| FlaF | CTCAGTTAAGTTAGTTTTTCAGGC |
| FlaR | GGTACTATGATGTTTGCTCTTAAATAG |
| InF | GTTAGAAGAATTATTCTTCACTAC |
| InR | GCTTTCATTATCTCATTGTAC |
| Lkd-spa-F | AAAGGAGTAGTTCCTCCATGTATAGAAAACATTTTAGGGAAGGA |
| Lkd-spa-R | TAGCTCCTTCCCTAAAATGTTTTCTATACATGGAGGAACTACTC |
| Lop-F | ACGCGTCGACATGGTATTAGACGTTAAGAAGTATCCT |
| Lop-R | ATAAGAATGCGGCCGCCTATTCTTTACTAAGGAAGTAGAGCTGC |
| S-BamHI-F | CGCGGATCCGATGGGGACTTTTACATTGCAC |
| S-NotI-R | AAGGAAAAAAGCGGCCGCTCATCTAACATAAGCCTTTAC |
| S-NdeI-F | GGGAATTCCATATGGGGACTTTTACATTGCAC |
| S-XhoI-R | CCGCTCGAGTCATCTAACATAAGCCTTTACCTCAC |
| L-NdeI-F | GGGAATTCCATATGGTATTAGACGTTAAGAAGTATCCT |
| L-XhoI-R | CCGCTCGAGCTATTCTTTACTAAGGAAGTAG |
| X-NdeI-F | GGGAATTCCATATGGTGAGCCAAGAGAAAAAAGTTAG |
| X-XhoI-R | CCGCTCGAGCTAGTTATTTTTTAATGCTTTCAT |
| Ln-Xc-BamHI-F | CGCGGATCCGATGGCATTAGACGTTAAAAAGTATCC |
| Ln-Xc-NotI-R | AAGGAAAAAAGCGGCCGCTTAGCTATTTTTTAATACTCTTATTATC |
| S-ove-SalI-F | ACGCGTCGACATGAACTGGAGCCATCCGCAGTTTG |
| S-ove-R | TCATCTAACATAAGCCTTTACCTCAC |
| L-ove-SalI-F | ACGCGTCGACATGGTATTAGACGTTAAGAAGTATCC |
| L-ove-R | CTATTCTTTACTAAGGAAGTAG |
| X-ove-SalI-F | ACGCGTCGACGTGAGCCAAGAGAAAAAAGTTAG |
| X-ove-R | CTAGTTATTTTTTAATGCTTTCAT |
| S-MLuI-R | CGACGCGTTCATCTAACATAAGCCTTTACCTCAC |
| Designation | Sequence (5’-3’) |
| L-SalI-F | ACGCGTCGACATGGTATTAGACGTTAAGAAGTATC |
| L-NotI-R | AAGGAAAAAAGCGGCCGCCTATTCTTTACTAAGGAAGTAG |
| S-qPCR-F | CGTGCAAGTTGACTGCTATGG |
| S-qPCR-R | TAGCTTGGAACGCCTACACC |
| L-qPCR-F | AGAGACGGATAGTACAAAAGGAGT |
| L-qPCR-R | ACTCAGCTTCCAATCCTCCC |
| X-qPCR-F | AAAAGTCGTCAGATTATTCGTGGA |
| X-qPCR-R | ACCAAGATTCATAAACTTTCCCAGA |
| Cdc6-2- qPCR -F | GCTATCTCTGATCTAGGGAAGGTA |
| Cdc6-2- qPCR -R | TCCGGTATATCAATTCCCTCTTCT |
| 16S-qPCR-F | GAATGGGGGTGATACTGTCG |
| 16S-qPCR-R | TTTACAGCCGGGACTACAGG |
| Sis-apt3-F | TACCCGGATCATATAACCCAG |
| Sis-apt3-R | AAGGTTTTTGTGGTTGGTGAT |
| Riol1spa-F | AAAGATTCTGGTTTACGAAAACATTCTTGTAATGGAATTTATTG |
| Riol1spa-R | TAGCCAATAAATTCCATTACAAGAATGTTTTCGTAAACCAGAAT |
| Riol1-DSB-F | AGCTAGAAGATTGAACATTGAGGAG |
| Riol1-DSB-R | CATAATCGCATCTTCGTAATCTATGG |
| dT32dC3 | TTTTTTTTTTTTTTTTTTTTTTTTTTTTTTTTCCC |
| dT32dG3 | TTTTTTTTTTTTTTTTTTTTTTTTTTTTTTTTGGG |
| dT30dC5 | TTTTTTTTTTTTTTTTTTTTTTTTTTTTTTCCCCC |
| IC-D25 | GCTAGGAATCTCTACACTGATCGTA |
| D25 | TACGATCAGTGTAGAGATTCCTAGC |

Table S2. Rates (%) of survival of various strains following treatment with DNA damaging agents

| Strain | MMS NQO |
| --- | --- |
| E233S  CHI-ΔX  PriL-kd  PriL-op | 16.66 ± 0.88 21.20 ± 0.77  9.88 ± 0.83 11.83 ± 0.69  10.20 ± 0.78 12.45 ± 0.76  36.40 ± 1.34 39.30 ± 1.49 |

Cells were grown to an OD_600_ of ~ 0.3 with shaking at 75°C, and treated for 6 h with 2 mM MMS or 2 μM NQO. Samples were plated for the determination of colony formation units (CFU). Survival rates were expressed as the percentage of surviving cells in drug-treated cultures relative to the corresponding untreated control cultures.

Table S3. Apparent mutation frequencies (×10^-7^) of various strains following treatment with DNA damaging agents

| Strain Untreated | MMS NQO |
| --- | --- |
| E233S 3.50 ± 0.33  CHI-ΔX 3.25 ± 0.38  PriL-kd 2.81 ± 0.61  PriL-op 4.20 ± 0.78 | 18.20 ± 1.22 26.70 ± 1.78  13.55 ± 1.76 20.40 ± 1.98  12.37 ± 1.23 19.44 ± 1.65  32.30 ± 1.69 53.70 ± 2.19 |

Cells were grown to an OD_600_ of ~0.3 with shaking at 75°C, and treated for 6 h with 2 mM MMS or 2 μM NQO. A sample of the culture was diluted and dilutions were plated on a plate containing 120 μM 6-methyl purine (6-MP) and 0.5 mM guanosine monophosphate (GMP). The apparent mutation frequency was calculated by first dividing the number of colonies on the 6-MP/GMP-containing medium by that on the medium lacking the mutagen, and then dividing the derived number by the dilution factor for the control.

Table S4. Proportions (%) of various types of mutation in different strains following treatment with DNA damaging agents

| Type of mutation | E233S, Untreated | E233S, MMS | E233S, NQO |
| --- | --- | --- | --- |
| GC→AT | 36.70 | 63.41 | 65.21 |
| AT→GC | 5.33 | 2.44 | 1.45 |
| GC→TA | 6.67 | 4.88 | 4.35 |
| GC→CG | 0 | 0 | 4.35 |
| AT→TA | 4.00 | 4.88 | 0 |
| Indels | 44.65 | 19.51 | 21.74 |
| Others | 2.65 | 4.88 | 2.90 |
| Type of mutation | CHI-ΔX, Untreated | CHI-ΔX, MMS | CHI-ΔX, NQO |
| GC→AT | 41.50 | 42.86 | 58.80 |
| AT→GC | 7.54 | 4.08 | 5.88 |
| GC→TA | 10.37 | 4.08 | 7.84 |
| GC→CG | 0 | 2.04 | 0 |
| AT→TA | 5.66 | 6.12 | 3.92 |
| Indels | 31.13 | 30.41 | 19.61 |
| Others | 3.80 | 10.41 | 3.95 |
| Type of mutation | PriL-op, Untreated | PriL-op, MMS | PriL-op, NQO |
| GC→AT | 37.86 | 32.38 | 32.23 |
| AT→GC | 6.83 | 5.71 | 7.43 |
| GC→TA | 8.95 | 7.62 | 9.10 |
| GC→CG | 0 | 0 | 4.13 |
| AT→TA | 5.31 | 7.62 | 1.65 |
| Indels | 34.38 | 36.19 | 37.19 |
| Others | 6.67 | 10.48 | 8.27 |

~100 colonies for each strain were picked from the plates obtained in the experiments shown in Table S3. The *apt3* gene as well as its promoter region in the genome were amplified by colony PCR, and the PCR products were sequenced. Mutations were identified, and the proportion of each type of mutation was calculated.

Table S5. Apparent mutation frequencies (×10^-7^) of various mutations in different strains following treatment with DNA damaging agents

| Type of mutation | E233S, Untreated | E233S, MMS | E233S, NQO |
| --- | --- | --- | --- |
| GC→AT | 1.285 ± 0.121 | 11.541 ± 0.774 | 16.690 ± 1.161 |
| AT→GC | 0.187 ± 0.018 | 0.444 ± 0.030 | 0.387 ± 0.026 |
| GC→TA | 0.233 ± 0.022 | 0.888 ± 0.060 | 1.161 ± 0.077 |
| GC→CG | 0 | 0 | 1.161 ± 0.077 |
| AT→TA | 0.140 ± 0.013 | 0.888 ± 0.060 | 0 |
| Indels | 1.563 ± 0.147 | 3.551 ± 0.238 | 5.805 ± 0.387 |
| Others | 0.093 ± 0.009 | 0.888 ± 0.060 | 0.774 ± 0.052 |
| Type of mutation | CHI-ΔX, Untreated | CHI-ΔX, MMS | CHI-ΔX, NQO |
| GC→AT | 1.349 ± 0.158 | 5.808 ± 0.754 | 11.995 ± 0.970 |
| AT→GC | 0.245 ± 0.028 | 0.553 ± 0.072 | 1.200 ± 0.970 |
| GC→TA | 0.337 ± 0.039 | 0.553 ± 0.072 | 1.599 ± 0.155 |
| GC→CG | 0 | 0.277 ± 0.036 | 0 |
| AT→TA | 0.184 ± 0.022 | 0.829 ± 0.108 | 0.800 ± 0.078 |
| Indels | 1.012 ± 0.118 | 4.121 ± 0.535 | 4.000 ± 0.388 |
| Others | 0.124 ± 0.014 | 1.411 ± 0.183 | 0.806 ± 0.078 |
| Type of mutation | PriL-op, Untreated | PriL-op, MMS | PriL-op, NQO |
| GC→AT | 1.590 ± 0.295 | 10.459 ± 0.547 | 17.308 ± 0.706 |
| AT→GC | 0.287 ± 0.053 | 1.873 ± 0.096 | 3.990 ± 0.163 |
| GC→TA | 0.376 ± 0.070 | 2.461 ± 0.129 | 4.887 ± 0.199 |
| GC→CG | 0 | 0 | 2.218 ± 0.090 |
| AT→TA | 0.223 ± 0.041 | 2.461 ± 0.129 | 0.886 ± 0.036 |
| Indels | 1.444 ± 0.268 | 11.689 ± 0.612 | 19.971 ± 0.814 |
| Others | 0.280 ± 0.052 | 3.385 ± 0.177 | 4.441 ± 0.181 |

The mutation frequency of a given type of mutation was calculated by multiplying the frequency of total apparent mutations (Table S3) by the proportion of this specific type of mutation (Table S4).

Table S6. Survival rates (%) of *S. islandicus* strains overproducing various primase subunits in the presence of CRISPR-generated DSBs

| Strain | Survival rate |
| --- | --- |
| pSe-Rp_DSB  pSe-Rp_DSB_PriS  pSe-Rp_DSB_PriL  pSe-Rp_DSB_PriX  pSe-Rp_DSB_PriSL | 1.60 ± 0.32  1.82 ± 0.48  5.05 ± 1.05  2.37 ± 0.56  8.13 ± 1.23 |

Strain E233S cells were transformed by electroporation with plasmid pSe-Rp_DSB_PriS, pSe-Rp_DSB_PriL, pSe-Rp_DSB_PriX or pSe-Rp_DSB_PriSL and plated for colony counting. The rate of survival was calculated by dividing the number of colonies from the cells transformed with the above plasmid by that from the control cells transformed with a control plasmid lacking the protospacer.

Table S7. Proportions (%) of different mutation types in *S. islandicus* strains overproducing various primase subunits in the presence of CRISPR-generated DSBs

| Strain | GC  **↓**  AT | AT  **↓**  GC | GC  **↓**  TA | GC  **↓**  CG | AT  **↓**  CG | AT  **↓**  TA | Indels |
| --- | --- | --- | --- | --- | --- | --- | --- |
| pSe-Rp_DSB (n=122, N=374) | 8.82 | 16.04 | 8.56 | 12.83 | 10.43 | 5.62 | 37.70 |
| pSe-Rp_DSB_PriS (n=117, N=371) | 11.32 | 14.56 | 8.63 | 15.36 | 7.28 | 4.85 | 38.00 |
| pSe-Rp_DSB_PriL (n=117, N=365) | 12.33 | 6.58 | 2.46 | 2.46 | 5.48 | 3.29 | 67.40 |
| pSe-Rp_DSB_PriX (n=120, N=369) | 10.57 | 17.61 | 9.76 | 13.28 | 3.25 | 4.88 | 40.65 |
| pSe-Rp_DSB_PriSL (n=120, N=481) | 12.47 | 1.67 | 2.49 | 1.67 | 2.08 | 0.62 | 79.00 |

Colonies were picked from the plates obtained from the experiments shown in Fig. 3A. The 40-bp spacer region was sequenced. Mutations were identified, and the proportion of each type of mutation was calculated. n, number of colonies analyzed. N, total number of mutations detected.

Table S8. Survival rates (%) of synchronous E233S cells following treatment with NQO

| Time after synchronization  treatment (h) | Survival rate |
| --- | --- |
| 0  1  2  3  4  5  6 | 93.70 ± 5.15  78.35 ± 3.50  88.43 ± 4.18  76.88 ± 3.90  1.60 ± 0.30  1.90 ± 0.40  80.81 ± 3.88 |

Strain E233S was grown at 75^o^C with shaking to an OD_600_ of ~0.2, synchronized by treatment with 6 mM acetic acid, and released into fresh medium. Samples were taken at intervals, treated for 15 min with 2 μM NQO, diluted, and plated for colony counting. The survival rates were obtained by dividing the number of colonies from the NQO-treated sample by that from the untreated control sample.

**Figure S1. Construction and growth of CHI-ΔX.**

(A) Design of CHI-ΔX. Numbers indicate the positions of amino acid residues in the proteins. The red star indicates the iron-sulfur cluster.

(B) Structure of the PriSLX complex predicted with AlphaFold on the basis of the partially resolved complex structure (PDB ID: 5OF3) with the portions of PriL and PriX deleted in the fusion protein shown in red circle (1, 2).

(C) Growth curves. Strain E233S and CHI-ΔX were grown with shaking at 75℃ or 90℃ and the OD_600_ values of the cultures were measured at indicated time points. All data points are an average of three independent measurements.


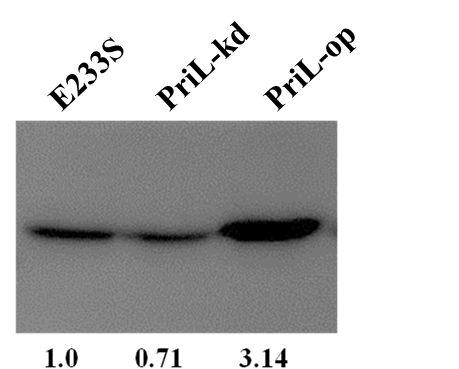


**Figure S2.** **Comparison of the protein levels of PriL in PriL-kd, PriL-op and the parental strain.**

PriL-kd, PriL-op and strain E233S were grown with shaking to an OD_600_ of ~0.6, and the cells were harvested and subjected to immunoblotting with antibodies against PriL. The fold changes in PriL level are indicated.

**Figure S3. Growth of various *S. islandicus* strains following exposure to IR.**

Cells were grown with shaking at 75^o^C to a OD_600_ of 0.005. The cultures were subjected to IR at dose of 800 Gy. The treated cells were inoculated into pre-heated medium, and grown with shaking at 75^o^C. The OD_600_ values of the cultures were measured at indicated time points. All data points are an average of three independent measurements.

**Figure S4. Oligonucleotide binding by PriSL, PriSLn-Xc and PriSLX.**

Primase subunits were incubated with 4 nM ^32^P-labeled 35-nt oligonucleotide dT32dC3, and samples were subjected to non-denaturing PAGE (8% polyacrylamide). The gel was exposed to X-ray film. Protein concentrations (μM): 0, 0.1, 0.4, 1.6 and 6.4.

**References**

1. Holzer, S., Yan, J., Kilkenny, M.L., Bell, S.D. and Pellegrini, L. (2017) Primer synthesis by a eukaryotic-like archaeal primase is independent of its Fe-S cluster. *Nat Commun* **8**, 1718.

2. Jumper, J., Evans, R., Pritzel, A., Green, T., Figurnov, M., Ronneberger, O., Tunyasuvunakool, K., Bates, R., [Žídek](https://pubmed.ncbi.nlm.nih.gov/?term=%22%C5%BD%C3%ADdek%20A%22%5BAuthor%5D), A., Potapenko, A. *et al*. (2021) Highly accurate protein structure prediction with AlphaFold. *Nature* **596**, 583-589.
